# The accuracy of several multiple sequence alignment programs for proteins

**DOI:** 10.1186/1471-2105-7-471

**Published:** 2006-10-24

**Authors:** Paulo AS Nuin, Zhouzhi Wang, Elisabeth RM Tillier

**Affiliations:** 1 Division of Cancer Genomics and Proteomics, Ontario Cancer Institute, University Health Network, 101 College St, M5G 1L7, Toronto, Ontario, Canada; 2 Dept. Medical Biophysics, University of Toronto, Toronto, Ontario, Canada

## Abstract

**Background:**

There have been many algorithms and software programs implemented for the inference of multiple sequence alignments of protein and DNA sequences. The "true" alignment is usually unknown due to the incomplete knowledge of the evolutionary history of the sequences, making it difficult to gauge the relative accuracy of the programs.

**Results:**

We tested nine of the most often used protein alignment programs and compared their results using sequences generated with the simulation software Simprot which creates known alignments under realistic and controlled evolutionary scenarios. We have simulated more than 30000 alignment sets using various evolutionary histories in order to define strengths and weaknesses of each program tested. We found that alignment accuracy is extremely dependent on the number of insertions and deletions in the sequences, and that indel size has a weaker effect. We also considered benchmark alignments from the latest version of BAliBASE and the results relative to BAliBASE- and Simprot-generated data sets were consistent in most cases.

**Conclusion:**

Our results indicate that employing Simprot's simulated sequences allows the creation of a more flexible and broader range of alignment classes than the usual methods for alignment accuracy assessment. Simprot also allows for a quick and efficient analysis of a wider range of possible evolutionary histories that might not be present in currently available alignment sets. Among the nine programs tested, the iterative approach available in Mafft (L-INS-i) and ProbCons were consistently the most accurate, with Mafft being the faster of the two.

## Background

The determination of homologous regions of molecular sequences is often used for the further inference of their function and evolution, and therefore accurate multiple sequence alignment (MSA) of nucleic acid and protein sequences is crucial. Consequently, there has been tremendous effort in the development and implementation of different MSA algorithms, using distinct approaches to improve the resulting alignment accuracy.

The accuracy assessment of MSA programs is often done by employing manually (or semi automatically) curated sequence databases such as BAliBASE [1], PREFAB [2] and SABmark [3]. So far, BAliBASE has been the most often used alignment database in evaluating the performance of different MSA programs. It was constructed using protein sequences or models with known three-dimensional structures. The last inception, version 3.0, had an increase in the number of available sequences and alignments. Such improvements apparently have addressed the major concerns of Karplus and Hu [4] regarding the use of BAliBASE to benchmark MSA algorithms.

Alignment databases provide a source of accurate alignments to gauge the accuracy and speed of different programs, but they also present several disadvantages. Even though the databases' alignments are manually curated, there is still the possibility of misalignments which would result in accuracy assessment problems. The sets of alignments still remain rather small and may not represent the complete range of scenarios of protein evolution. Furthermore, a major drawback of the use of alignment databases is that algorithms can potentially be developed and tuned to the alignments present solely in these data sets.

Recently there have been several DNA sequence simulation packages that incorporate indels, such as MySSP [5] and DAWG [6]. MySSP has been widely used in different studies of phylogenetic inference and evolutionary distance estimation coupled with DNA alignment accuracy [7,8]. For proteins, Lassmann and Sonnhammer [9] in a previous comparison of MSA algorithms used artificially created sequence sets generated by the simulation program Rose [10]. Rose simulates sequences of proteins allowing for the occurrence of indels. Data sets generated by Rose present their own limitations for the study of the alignment accuracy. In Rose, indel size and number do not adequately represent empirical data for proteins that have diverged for different evolutionary times. Also the program assumes equal evolutionary rates of all the sites in the protein.

In this study we introduce an improved approach to assess alignment accuracy by using simulated protein sequences generated by Simprot [11]. Simprot is an advanced simulation program that employs a parameterized version of the Qian and Goldstein [12] insertion and deletion (indel) distribution. Although the original distribution was empirically derived from a subset of alignments of highly diverged protein sequences, the parameterized version permits a very flexible simulation of indels in sequences for all levels of sequence divergence. Simprot also allows variable substitution and indel rates at different sites by implementing gamma distributed sites rates [13]. Three models of amino acids substitution (PMB, PAM and JTT) are also available. We have used Simprot to generate known alignments with a wide variety of evolutionary parameters, as well as the latest BAliBASE database of curated alignments, to investigate the accuracy and speed of popular and publicly available protein multiple sequence alignment software programs.

### Alignment programs

There are many available computer packages that generate MSAs of protein sequences. We selected nine of the currently most often used programs (in order of publication date): Clustal W, Dialign2.2, T-Coffee, POA, Muscle, Mafft, ProbCons, Dialign-T and Kalign.

#### Clustal W [14] version 1.8

This is probably the most widely used alignment program and oldest among the packages tested. The software performs a progressive alignment, first employing a pairwise sequence comparison by calculating a distance matrix that stores sequence divergence. After this matrix is obtained, a tree guide is built using Neighbor Joining, followed by the third and final step where sequences are aligned according to the branch order in the guide tree. The program employs two gap penalties in its alignment procedure: gap opening and gap extension, and in the case of polypeptides, a full amino acid scoring weight matrix. These gap penalties are mainly dependent on factors such as the weight matrix, sequence length and similarity. In simple cases, Clustal W might accurately align corresponding domains and sequences of known secondary or tertiary structure while in more complex cases it can be used as a good starting point for further refinement.

#### Dialign2.2 [15] version 2.2.1

This program uses a *diagonal *method to align sequences locally and globally. Dialign2.2 does not compare single residues, but whole uninterrupted (no gaps, mismatches allowed) stretches of residues that would form diagonals in a dot-matrix comparison of two sequences. Consequently, it does not penalize the insertion and extension of gaps, and may leave unrelated segments unaligned. The first step in the procedure creates all possible pairwise alignments, storing a collection of *diagonals *meeting certain consistency criteria [16] without conflicting double or crossover assignments of residues [15]. All saved *diagonals *are weighted in order to define entries with maximum sum of weights, and then sorted in order to determine the degree of overlap, emphasizing the existence of *diagonals *present in multiple sequences. A greedy-like algorithm does a final processing, checking *diagonals *scores from top to bottom creating a final multiple alignment. Gaps are inserted at the end of the MSA creation until all present residues are connected.

#### T-Coffee (Tree-based consistency objective function for alignment evaluation) [17] version 3.27

T-Coffee employs a progressive strategy in aligning sequences. The program first creates a library from two different sources: global alignments from Clustal W and local alignments from Lalign [18]. For each pair of sequences global alignments and the pairwise local alignments are created from the ten top-scoring non-intersecting segments. The program processes the global and local information, assigning weights to all pairwise alignments relative to sequence identity [19]. This is followed by the combination of groups that are merged into a single library. There is an extension phase for this combined library, making the final weight of any pair of residues reflect part of the information contained in the whole library. A final step requires a calculation of a distance matrix and a Neighbor Joining tree, since the alignment is generated with a progressive strategy by aligning the two closest sequences on the tree according to the weight stored in the extended library. The initial pair is then fixed and any existing gaps cannot be shifted later. The progressive alignment continues until every sequence is aligned.

#### POA (Partial Order Alignment) [20] version 2.0

POA is another MSA package that uses a progressive alignment algorithm without using generalized profiles. This program introduces the use of a Partial Order-Multiple Sequence Alignment (PO-MSA) format to represent sequences, and more accurately reflects biological content. This format stores the alignment as a compacted graph for minimal node and edge counts, still containing all the information available in a traditional MSA. Sequences are stored as a linear series of nodes each connected by two edges. POA uses a traditional dynamic programming algorithm [21,22], where linear sequences are replaced by Partial Order (PO) graphs. These PO structures are transformed in usual 2D matrices and each combination of cells are scored backwards as in a traditional Smith-Waterman sequence alignment procedure [22]. These matrices are then extended in any direction (diagonal, horizontal, vertical) allowing the production of the pairwise alignment on junction points. The MSA is obtained from the alignment of two sequences at the beginning with the addition of other sequences successively to the initial pair.

#### Muscle (Multiple sequence comparison by log-expectation) [2,23] version 3.6

Muscle uses a pairwise profile alignment approach. The program first builds a progressive alignment which is then improved and refined in two subsequent stages. The progressive alignment is created after the sequence similarities, a distance estimation and a UPGMA tree are calculated. Muscle uses two distance measures: a *k*mer distance for unaligned sequence pairs and a Kimura distance for aligned pairs [2]. The progressive alignment improvement stage creates a new tree with the already calculated Kimura distance matrix and then builds a better alignment based on this ameliorated tree. The last refinement stage employs a variant of the tree dependent restricted partitioning [24]. This method deletes one of the tree edges, bi-partitioning the alignment and extracting both partitions' profiles which are then realigned with a profile-profile alignment. Every tree edge is visited iteratively and the alignment with an updated summed pairwise score of each sequence pair is retained. The edges are visited in order of decreasing distance from the root, with a realignment of individual sequences, moving to more closely related groups of sequences [23].

#### Mafft (Multiple sequence alignment based on Fast Fourier Transform) [25] version 5.732

Mafft is a program that can be used with different alignment approaches, either progressive alignment alone (with Fast Fourier Transform), or progressive followed by iterative refinement. Mafft's basic run can have up to three steps, but the default procedure performs the initial two steps. First, a progressive alignment is created based on a rough distance between every sequence pair based on shared 6-tuples. A guide tree is also generated by UPGMA with modified linkage and sequences are then aligned following the branch order of the tree (this step alone is called strategy FFT-NS-1). The second step recalculates a distance matrix, based on the information gathered on the previous step, and the progressive alignment is re-done using a tree obtained from the new matrix as a starting point (up to this step, the strategy is known as FFT-NS-2 and it is the default used by the software). The last phase is the iterative refinement which optimizes the Gotoh's weighted sum of pairs (WSP) [26] score, with a group-to-group alignment [27] and the tree-dependent restriction partition technique [24]. If all three steps are employed, the procedure is called FFT-NS-i, meaning it uses an FFT method to rapidly identify homologous regions present in the sequences which is followed by an iterative phase of refinement. FFT converts every single amino acid present in a sequence to a vector representing volume and polarity, which are important factors on substitution events, allowing the software to predict such occurrences with precision.

Mafft also includes three additional refinement algorithms: L-INS-i, G-INS-i and E-INS-i [25]. These strategies increase the number of steps required to create an MSA alignment to five. In these cases the first step also requires the construction of a distance matrix, not using 6-tuples. Differently from the FFT-NS-* approaches there is no reconstruction of the calculated UPGMA tree and the program moves to the second step, dividing the gap-free segments and storing score arrays for each gap-free segment from one sequence to another. Mafft then calculates an "importance" value from the score of the segment and stores how frequently residues appear on other segments. All "importance" values are then gathered in an "importance" matrix in step three which is quickly followed by a group-to-group alignment obtained from the score matrices and a weighting scheme [14] based on a Needleman-Wunsch algorithm. A final step iteratively refines the obtained alignments, optimizing a WSP score and the "importance" values calculated previously.

#### ProbCons (Probabilistic Consistency-based multiple sequence alignment) [28] version 1.1

ProbCons is the only program that uses a probabilistic consistency method of alignment. It is a modification of the traditional sum-of-pairs scoring system, and in addition incorporates a pair-hidden Markov model-based progressive alignment algorithm. The alignment procedure is divided into four steps, starting with a computation of posterior-probability matrices for every pair of sequences. This is followed by a dynamic programming calculation of the expected accuracy of every pairwise alignment. Probabilistic consistency transformation is then employed in order to re-estimate the match accuracy scores. A guide tree is calculated with hierarchical clustering with the similarity defined by a weighted average of values between sequences of each cluster. The guide tree is used to align the sequences using a progressive approach. A post-processing phase is also done, where random bi-partitions of the generated alignment are realigned in order to check for better alignment regions. ProbCons differs from other alignment programs since it does not incorporate biological concepts such as position-specific gap scoring, evolutionary tree construction and other features commonly used by other packages.

#### Dialign-T [29] version 0.2.1

This program is a re-implementation of the procedure developed in Dialign2.2, but with a better solution to deal with inconsistent fragments, including fragment-chaining. It also implements a new approach for estimating probabilities of the random occurrence of each fragment present in the sequence to be aligned. Dialign-T does not use pre-calculated tables in order to obtain weight scores: it calculates probability tables from several substitution matrices. Additionally, the greedy-like multiple alignment algorithm from Dialign2.2 was changed in order to avoid spurious local similarities.

#### Kalign [30] version 1.04

Kalign is another program that uses a progressive alignment approach to obtain the best MSA possible. The main difference of this algorithm to other methods is that it employs the Wu-Manber approximate string matching algorithm [31] when calculating the distance among sequences. The Wu-Manber algorithm measures the distance between two strings using a Levenshtein edit distance, which allows an efficient search for mismatches (shared or not) and patterns present in the sequences. According to the Kalign developers, this methodology allows for a distance estimation which is as fast as an *k*-tuple algorithm but is more accurate [30]. The first step in the alignment procedure is to calculate the pairwise distances using the Wu-Manber approach. The pairwise distance estimation is followed by a construction of a guide tree by using UPGMA, which is employed in a global dynamic programming method to align the sequences/profiles. Additionally, the program performs a consistency check in order to define the largest set of sequence matches that can be inserted in the alignment, using a modified version of the Needleman-Wunsch [21] to find the most consistent path through the dynamic programming matrix. Also, Kalign updates the positions of pattern matchings, which adjusts the absolute position of matches found within sequences to their relative positions within generated profiles [30].

## Results

### Simprot simulated sequences

Simprot's simulation parameters provide flexibility for generating alignments so that the effects of distinct factors can be examined together and/or separately under multiple evolutionary scenarios. Simulated sequences were used to investigate the influence of sequence length, indel frequency, indel length, evolutionary distance, terminal gaps length, gamma density function and tree topology on the accuracy of alignments inferred by different programs. More than 30000 alignments were created independently by Simprot using five phylogenetic trees (Figure 2) with variable lengths and different number of variable size indels, in order to cover different topological evolutionary patterns. Simprot generates a known alignment and another file containing the sequences with no indels. One hundred simulated alignments with different random seed values were created for each combination of tested parameters. All corresponding sequences were also aligned with the nine programs described above and the resulting alignments were compared to the "true" alignment generated by Simprot. The average accuracy values for the 100 alignments of each set are reported here and in some cases a Wilcoxon signed ranks test was employed in order to determine the statistical significance of the difference on average accuracy. The protein substitution matrix used in all simulations was PMB [32], which is also the program's default.

**Figure 1:**
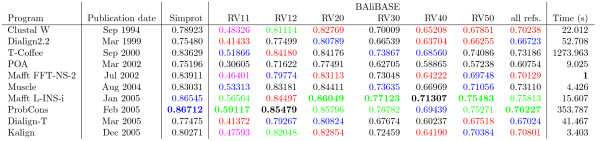
**Overall average accuracy values obtained with all Simprot's simulated sequences and all BAliBASE's references**. Results are ordered by date of publication. Values in the same column that are not significantly different according to a Wilcoxon signed ranks test (*p *< 0.05) have the same colour; values in black are significantly different, and bold font represents the largest value in the column. CPU times are normalized to Mafft FFT-NS-2 and were obtained with a 44 sequence alignment of 500 residues.

**Figure 2:**
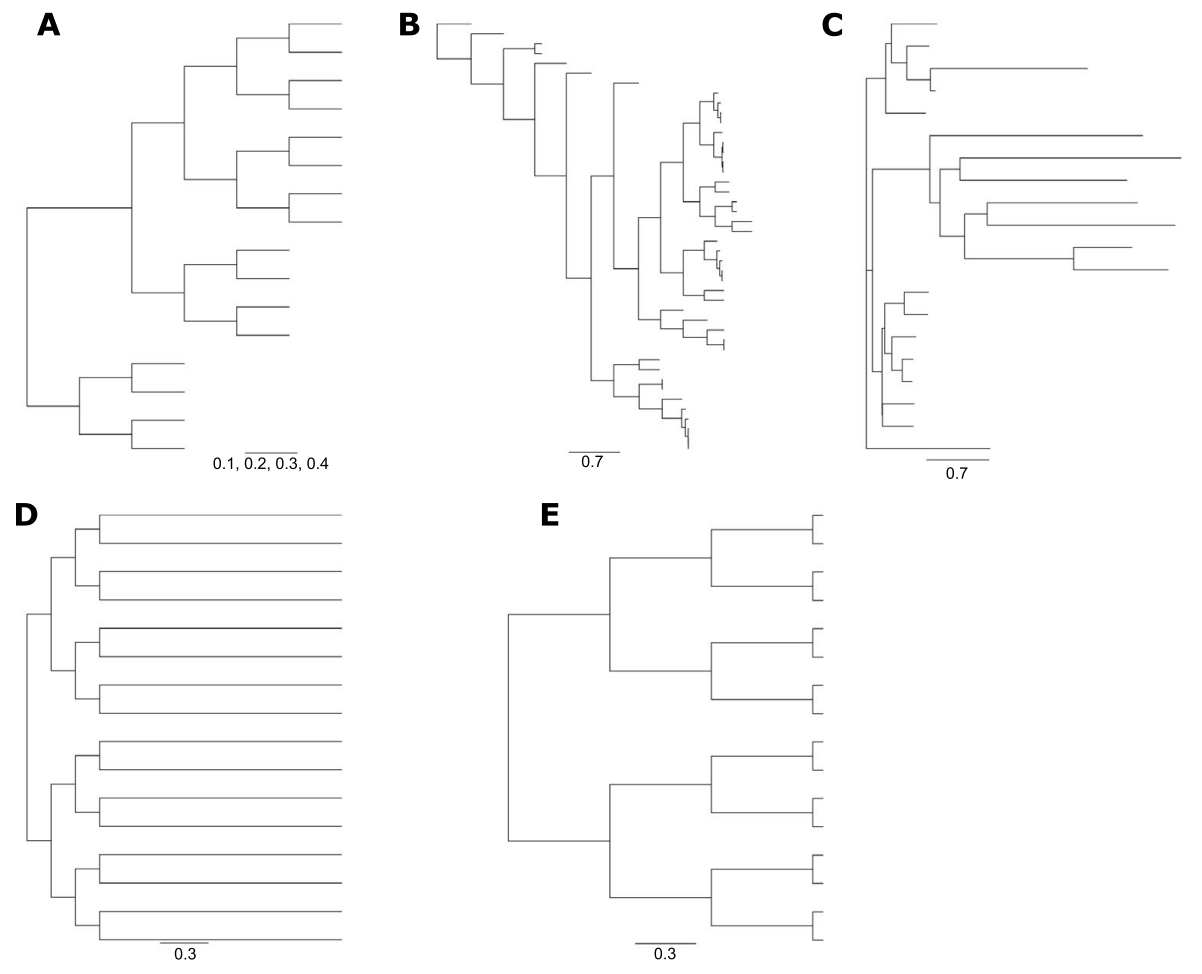
**Tree topologies used in the analysis**. **A**, **D **and **E **are artificially created topologies, while **B **and **C **are based on PFAM alignments.

**Figure 3:**
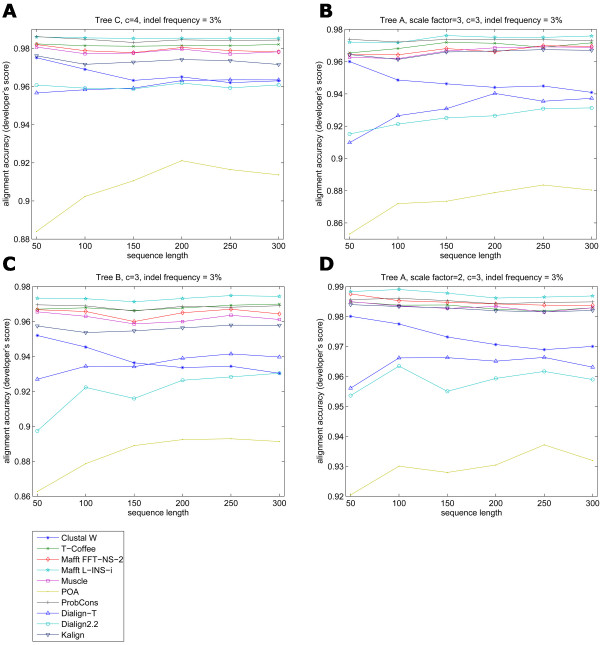
**Comparison of alignment accuracy and increasing sequence length, at low indel frequency values. Selected examples with different input trees**. The increase in sequence length did not seem to affect alignment accuracy of the majority of the programs. ProbCons and Mafft L-INS-i were the top performers, followed closely by Muscle, T-Coffee, Mafft FFT-NS-2 and Kalign. Dialign2.2, Dialign-T and Clustal W presented a better accuracy than POA in most of the cases. Scale factor: value by which tree's branch lengths are multiplied, making them uniformly change; *c *is the Qian-Goldstein distribution value that determines average length of indels.

**Figure 4:**
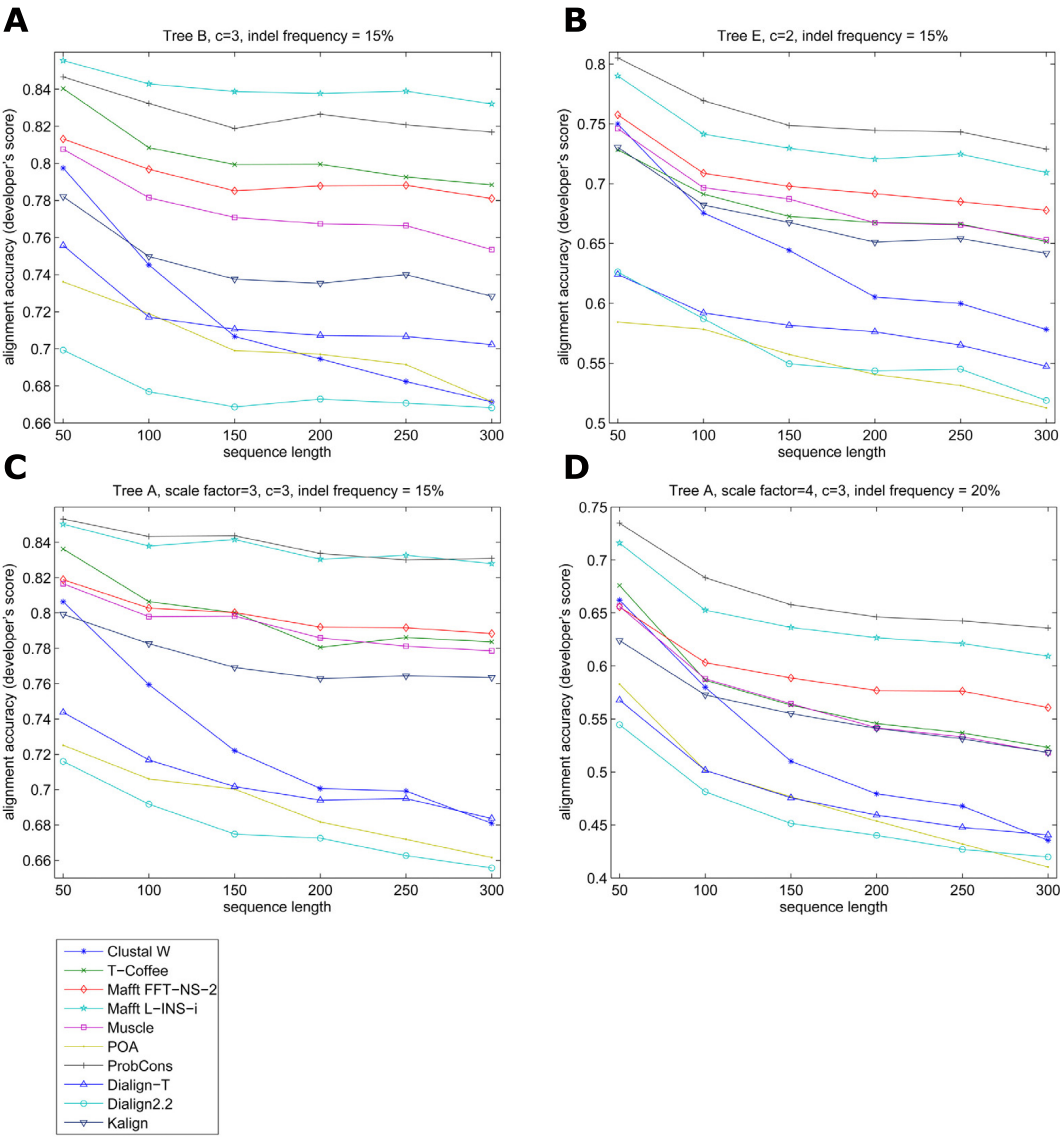
**Comparison of alignment accuracy and increasing sequence length, at high indel frequency values. Selected examples obtained with different tree topologies**. ProbCons and Mafft L-INS-i took turns as top performers. A middle group of programs is revealed by this comparison, comprising Mafft FFT-NS-2, T-Coffee, Muscle and Kalign. The smallest accuracies were shown by Dialign2.2, Dialign-T and POA, and Clustal W at large sequence sizes. Clustal W curves presented a steep decline in accuracy as the sequence length was increased.

As reported previously [9], sequence length does not affect alignment accuracy of the different programs. In order to confirm this, five different root sequence lengths were employed in the analysis: 50, 100, 150, 200, 250 and 300 amino acids. These values were selected in order to get the resulting alignments in a feasible amount of time, while maintaining a significant difference in root sequence lengths. To determine the effect of amino acid substitutions and sequence length on alignment accuracy, we first kept the indel frequency and indel length very low and considered different trees with various overall evolutionary distances and with increasing root sequence lengths. The majority of the packages tested generated results ranging from good to excellent with increasing sequence length. POA presented the lowest accuracies on the performed tests and at the same time was positively affected by sequence length increase (Figure 3). POA's lower accuracy appears to be due to a tendency of the program to place large internal gaps close to the sequence terminals, while the accuracy increase in larger sequence lengths might be explained by the proportionally small influence of these terminal gaps in the alignment scoring. Apparently, the alignment of sequences with a large number of substitutions but a low number of gaps did not present a problem for any of the nine algorithms used, no matter the size of the sequence. Noticeably, Clustal W showed the steepest decline in accuracy when sequence length was increased even at very low gap frequency values (Figure 3 and 4). The program occasionally had an alignment accuracy decrease two to three times larger than the average for all other seven programs (Figure 4B), especially when indels were added to the reference alignments.

Different indel frequencies were also used in the simulations in order to test the effect of indel occurrences in alignment accuracy. Simprot's process for insertions and deletions assumes a Poisson model, where the expected frequency of indels between two sequences separated by an expected 100 PAM distance is

*p *= 1 - *e*^-*z*/*c*^

where *z *is the indel probability that is scaled by the evolutionary scale factor *c*. The smallest frequency *p *employed was the program's default value 3% and increased up to 30%. As expected, when indels were added to the simulated sequences and evolutionary distance was increased, there was an evident loss in accuracy for all programs. This corroborates results obtained by Lassmann and Sonnhammer [9], who showed that programs tended to have poorer performance as the evolutionary distance increased when indels were present. The best results were generated by ProbCons and Mafft L-INS-i. ProbCons presented better results for trees with longer evolutionary distances when intermediate to large indel frequencies were applied. Conversely, Mafft L-INS-i performed better for smaller evolutionary distances and with intermediate indel frequency values (Figure 4).

**Figure 5:**
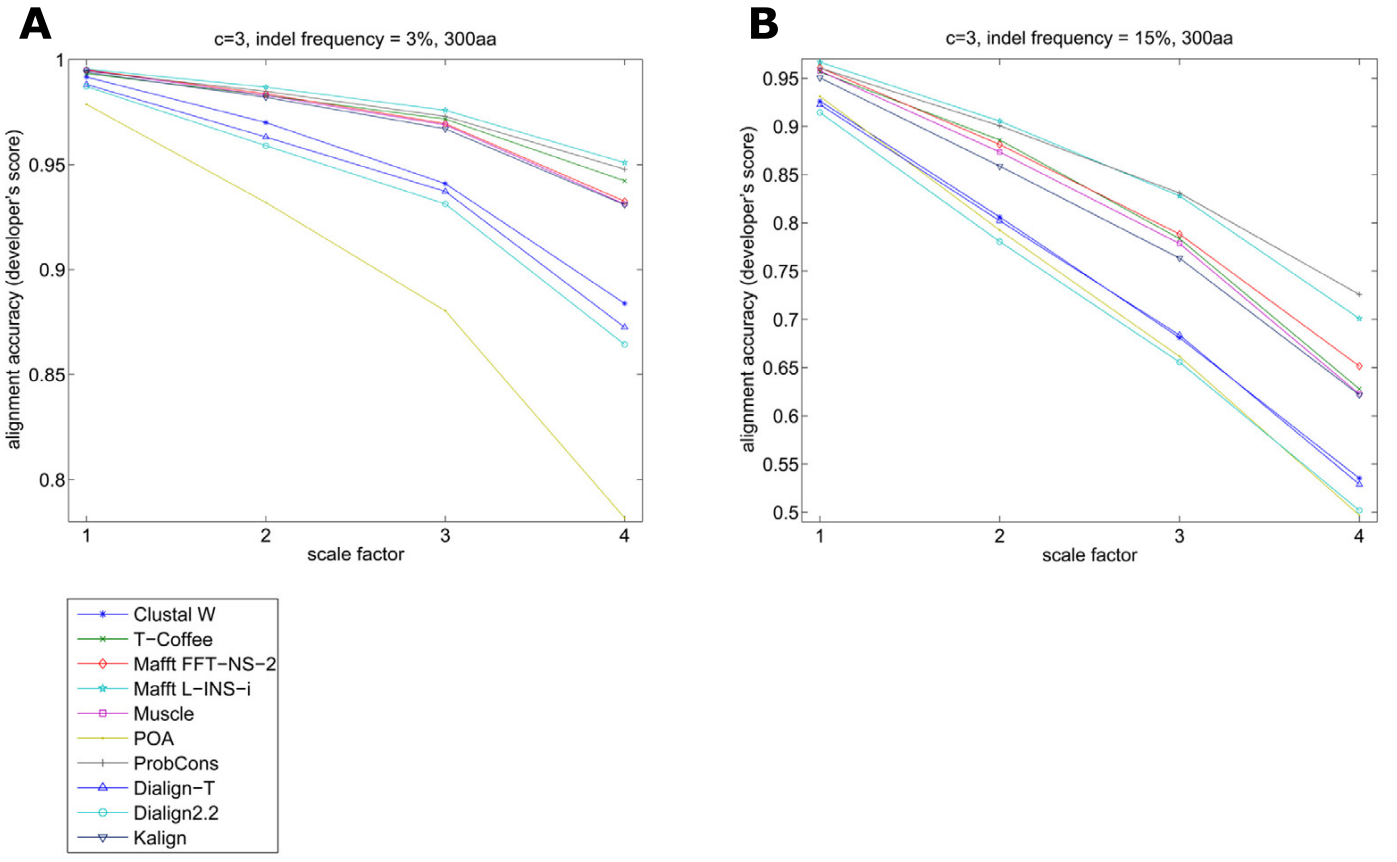
**Decrease in accuracy with an increase in the evolutionary scale factor of topology A**. POA seemed to be the most affected by the increase of the scale factor applied to topology A from Figure 1. The top performers are again Mafft L-INS-i and ProbCons. An intermediary group formed by T-Coffee, Muscle, Mafft FFT-NS-2 and Kalign is followed by Dialign2.2, Dialign-T, Clustal W and POA that showed poor accuracy values as the scale factor increased.

In Simprot the evolutionary distance set by the branch lengths in the input tree affects the expected number of substitutions and also affects the expected number of insertions and deletions. In order to further analyze the influence of branch lengths on alignment accuracy, we considered a single tree topology and scaled the branches (Figure 2, tree A) so that the overall tree shape was not changed (Figure 5). As shown above, all programs were negatively affected by increased evolutionary distances, particularly when the employed indel frequency parameter was high. POA had the steepest decline in accuracy, at small indel frequencies (Figure 5A).

**Figure 6:**
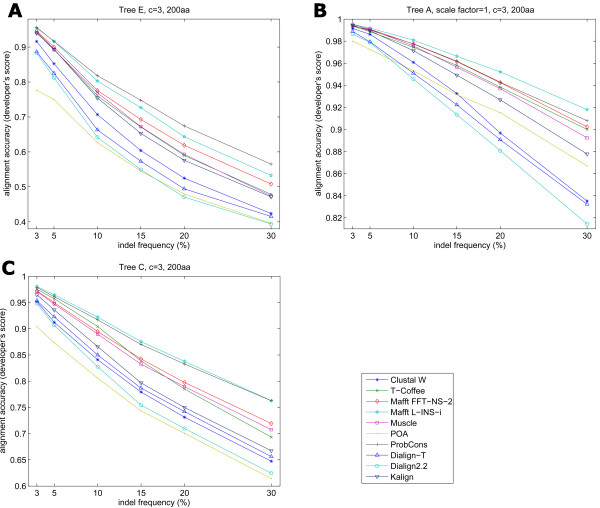
**Accuracy decline with larger indel frequency values**. Different accuracy values from alignments of sequences simulated with distinct topologies and increasing the indel frequency.

Due to the fact that indel frequency appeared to have a large effect on MSA accuracy, we analyzed the effect of increasing the indel frequency independently of other factors (Figure 6). Our results showed that accuracy and its rate of decline with indel frequency depended on the input tree used. Trees with longer branch lengths (Figure 6A) had sharp decreases in accuracy with increasing indel frequencies. Input trees with shorter branch lengths showed smaller declines in accuracy (Figure 6B–C). For most programs, when a topology with varied branch length was used, the accuracy decrease was almost linear with increasing indel frequency. ProbCons and Mafft L-INS-i were the least affected by the increase in evolutionary distance and resulted in the best performances.

**Figure 7:**
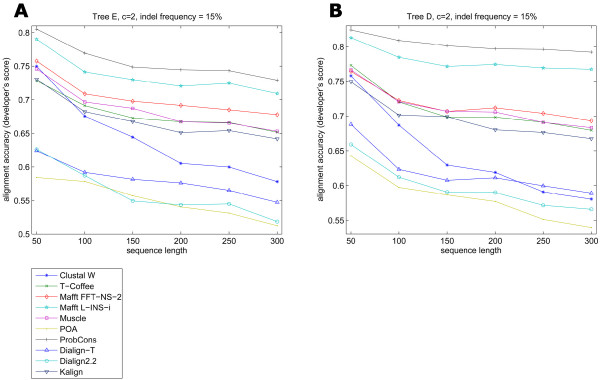
**Comparison of the alignment accuracy values of trees with the same maximum evolutionary distance and different topologies**. The accuracy curves for both topologies are very similar, independently of the topology employed. The input trees differed in where evolution had occurred, at the tips or internal branches.

Another element that can influence the occurrence and number of indels in protein sequences is the tree topology. We considered this independently of evolutionary distance and Simprot's indel frequency by considering two trees with identical maximum evolutionary distance (Figure 2, Trees D and E) but with different topologies. Although evolution had occurred at different locations in the two tree topologies tested (tips opposed to internal nodes), this did not seem to have large influence on overall alignment accuracy for the majority of the algorithms analyzed (Figure 7), as in both cases programs obtained similar alignment scores.

**Figure 8:**
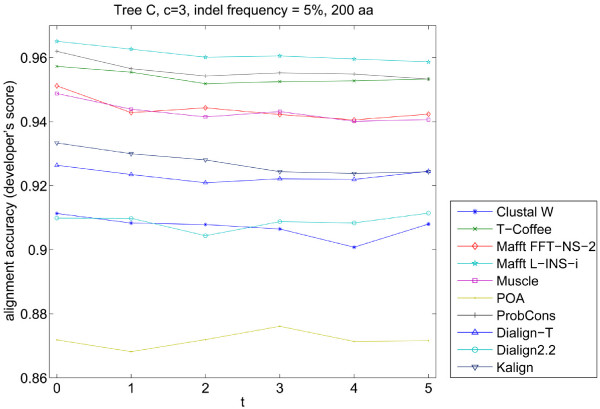
**Alignment accuracy comparison when increasing terminal gaps were inserted in the alignments**. The program rankings are much different than in other situations analyzed. Mafft L-INS-i and ProbCons were the top performers, followed closely by T-Coffee, Muscle and Mafft FFT-NS-2. *t *is the parameter used to determine the length of inserted terminal gaps in Simprot.

Real protein sequence data often contains non-homologous terminal ends and/or incomplete sequences. We investigated the effect of large terminal gaps on alignment accuracy. A small modification in Simprot's code was necessary to include an additional probability of terminal gaps. Since no reasonable biological model exist for external gaps, we introduced an *ad hoc *parameter *t *which determines the probability and length of external gaps by scaling the probabilities for internal gaps. Five different values of the terminal gaps insertion parameter were used while keeping the internal gap frequency constant (5%) for these simulations. It was observed that the presence of terminal gaps, regardless of their length, had a minimum effect on alignment accuracy for most of the programs (Figure 8). Again in this case, Mafft L-INS-i and ProbCons were the top performers.

**Figure 9:**
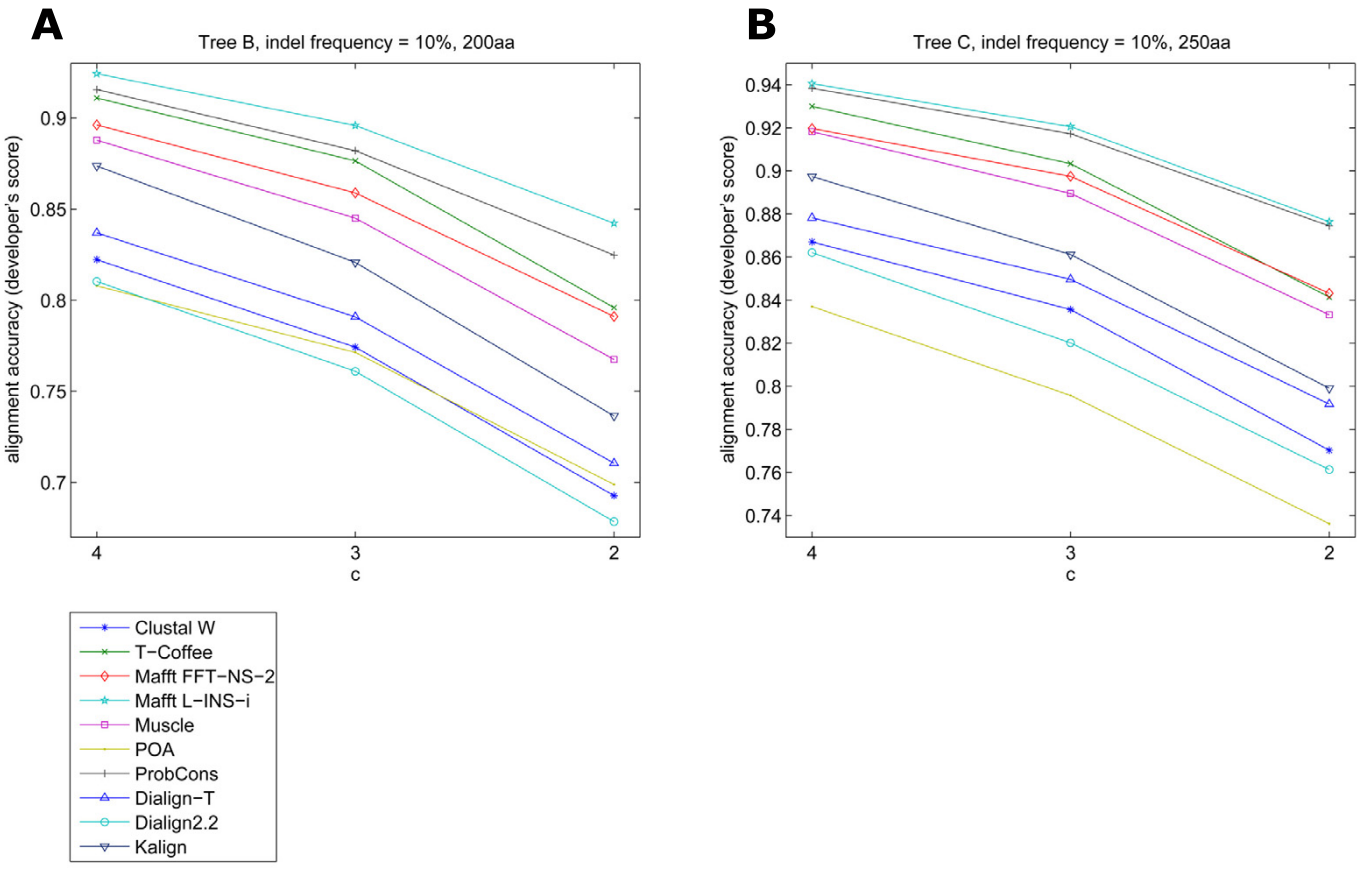
**Alignment accuracy compared to decreasing *c *values**. The lower the *c *value, the longer the indels. Both examples show the modest effect of longer indels on the alignment loss of accuracy. ProbCons and Mafft L-INS-i are the top performers, followed by T-Coffee, Mafft FFT-NS-2, Muscle and Kalign respectively. There is a bottom group formed by the remainder of the programs.

The analysis of influence of the indel frequency on alignment accuracy did not take into account the overall size of the simulated insertions and deletions. To test a possible effect of indel size on the programs' performances three different values (2, 3 and 4) of Simprot's *c *parameter were tested. This value is used by the generalized Qian and Goldstein distribution [11,12] in indel length determination. Larger *c *values yield shorter indels while smaller values result in longer ones [11]. The indel frequency was kept constant. We found that the larger the indels the lower the alignment accuracy, although with a moderate difference in the final average score (Figure 9). This accuracy loss could be seen for all phylogenetic trees analyzed and for the majority of the programs. Again, Mafft L-INS-i and ProbCons seemed to fare better and were least affected by the variation in gap length.

**Figure 10:**
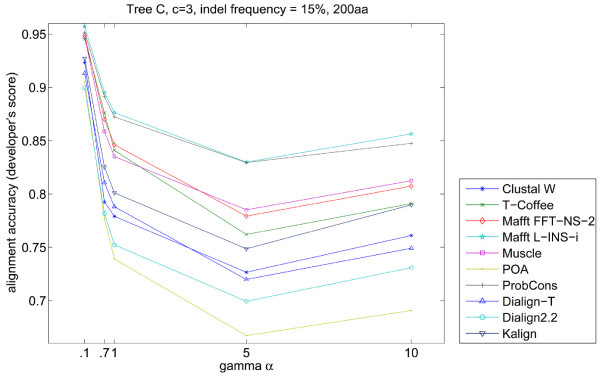
**Alignment accuracy compared to increasing gamma *α***. The programs' accuracy decreased with *α *values up to 5, reversed when the value increased to 10. POA is the most affected by the gamma *α *increase, while Mafft L-INS-i and ProbCons are the least affected.

According to Rosenberg [7], changes in the shape of the gamma distribution, of Yang's [13] distribution of evolutionary rates, influences alignment accuracy. The gamma shape models the proportion of slow to fast evolving positions and accounts for variable substitution rates among sites; the lower the *α *the larger the number of sites with low substitution rate. Decreasing gamma's *α *shape parameter positively affected the alignment accuracy (Figure 10), due to an increasing number of identical sites between sequences. Simprot allows the modification of the gamma's *α *shape parameter and in our study it was set at 0.1, 0.7, 1 (Simprot's default), 5 and 10. We examined changing *α *using different topologies and indel frequency values. The obtained results show a moderate influence of the value of gamma *α *at low indel frequencies, resulting in an accuracy loss for most of the programs, especially for POA. When larger indel frequencies were employed, the negative effect of increasing gamma was accentuated up to *α *= 5, which was reversed by a small gain in accuracy when *α *was increased from 5 to 10 (Figure 10). This is expected, since with *α *values the gamma distribution of evolutionary rates tends to be less extreme (exponential) and to have a curve shape similar to the normal distribution.

**Figure 11:**
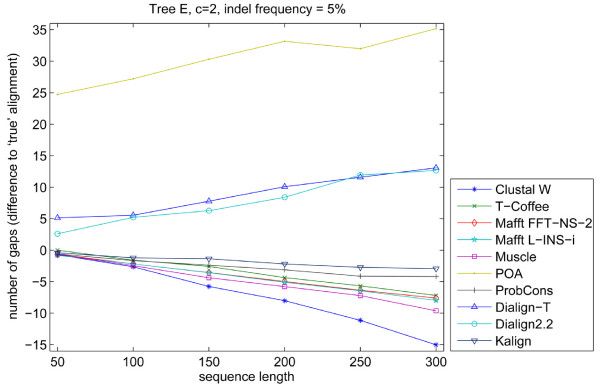
**Number of gaps inserted by programs relatively to the known alignment**. The curves show the difference in the number of gaps relative to the known alignment generated by Simprot. Kalign had the smallest difference for most of the sequence lengths, while POA was the program with the largest difference. Above zero are programs that do not generate a guide tree for multiple alignment, while below zero are the programs that do generate a guide tree.

In order to deduce why there was a large influence of insertions and deletions on the programs' performance, we analyzed the average number of gaps per sequence present in the "true" alignment and in all resulting alignments (Figure 11). As mentioned above, POA had the tendency of inserting long internal gaps at the sequence terminals; this inflates the average number of gaps in the alignments that are constructed by the program. Clustal W, under default parameters, was the most conservative of the programs tested and in every case had a smaller gap number average for its alignments than other packages and the known alignment. Kalign and ProbCons were the programs with a final number of inserted gaps closest to the real alignment in the majority of the simulations. Overall, programs that use a progressive alignment with tree determination showed a smaller gap number average per sequence than the programs that do not use a guide tree (POA, Dialign2.2 and Dialign-T).

In summary our results show that it is the total number of indels independently of where in the tree they occur, and to some degree independently of the number substitutions, that had the greatest effect on alignment accuracy. Also, indel size plays a role in alignment accuracy, but to a lesser extent than indel number. Additionally, the gamma distribution of evolutionary rates generally had a negative effect on the final accuracy. Regarding program performance, ProbCons and Mafft L-INS-i achieved the best results in the majority of the simulated alignments sets. An intermediary group consisted of T-Coffee, Muscle, Mafft FFT-NS-2 and Kalign, while Clustal W, POA, Dialign-T and Dialign2.2 often produced the poorest alignment accuracy. An overall summary of alignment accuracy for each program is shown in Figure 1. With the exception of Clustal W, in scenarios of large sequence lengths and indel frequency, programs that have a tree-guided multiple alignment procedure showed better results than those that do not rely on tree determination to align protein sequences. As pointed above, programs with a tree-determination step were more conservative in inserting gaps than programs that lack this step, generally achieving better final accuracies.

### BAliBASE

It was important to determine if the results obtained from the Simprot-generated sequences were applicable to alignments from actual proteins. We considered the accuracy of the nine programs on the latest version of BAliBASE alignments (Figure 1). Overall we found results similar to those obtained on the simulated sequences in that ProbCons and Mafft using strategy L-INS-i appeared to have the best performance. In BAliBASE's reference RV11, containing equidistant sequences sharing less than 20% identity, ProbCons and Mafft L-INS-i were not statistically different according to the Wilcoxon signed ranks test (p > 0.05). The same result with no statistical separation was observed in reference RV20, which is composed of sequences from divergent subfamilies, in reference RV30, comprised of sequences from protein families with some highly diverged sequences, and in reference RV50, made of sequences with large insertions.

ProbCons did perform significantly better than Mafft L-INS-i in the set RV12 that contains equidistant sequences sharing between 20 and 40% identity. Mafft L-INS-i and T-Coffee were not statistically different (Wilcoxon signed ranks test, p > 0.05). Conversely, on reference RV40 composed of protein sequences with large extensions, Mafft L-INS-i outperformed all other packages, with ProbCons and T-Coffee not far behind and not significantly different.

When results for all references are analyzed together, the same pattern observed from the isolated references was also found. In this broader scenario, ProbCons and Mafft L-INS-i achieved the best results and the difference in final alignment accuracy is not statistically significant (Wilcoxon signed ranks test, p > 0.05). An intermediary pack is formed of two distinct groups (defined by Wilcoxon signed ranks test) where Muscle and T-Coffee did slightly better than Mafft FFT-NS-2, Kalign and Clustal W. Showing the poorest performance for the whole database set were Dialign-T, Dialign2.2, which were statistically indistinguishable, and POA. Overall, the results from Simprot and BAliBASE data sets were consistent, with the exception of Mafft FFT-NS-2 which ranked significantly lower on BAliBASE data sets than on Simprot's. These results corroborate in part the findings of Lassmann and Sonnhammer [9], that showed T-Coffee as the best available algorithm at the time for BAliBASE v2 alignments. Their result also indicated POA as the program with the poorest performance.

### Speed of execution

**Figure 12:**
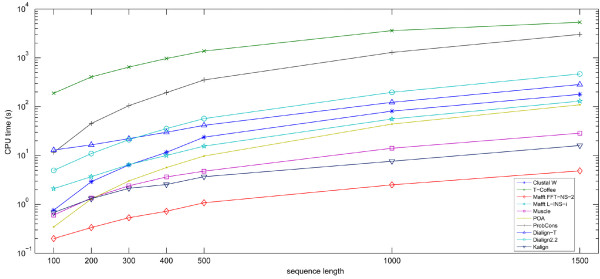
**CPU time spent by each program when aligning increasing sequence lengths**. Mafft FFT-NS-2 is clearly the fastest algorithm while T-Coffee is the slowest among all programs tested.

Mafft FFT-NS-2 was the fastest program for all tested sequence sizes (Figure 12). T-Coffee, as shown before [9], had the worst speed, with an average alignment time for the smallest sequence set (100 amino acids) longer than for Clustal W, Mafft (FFT-NS-2 and L-INS-i), Kalign and POA when aligning the largest set. ProbCons had the second worst average time for most sequence sizes.

## Discussion

Overall, Mafft L-INS-i and ProbCons generated the best alignments on our test data, including simulated sequences and BAliBASE's v3.0 reference sets, while POA, Dialign2.2, Dialign-T and Clustal W had the worse accuracy. The intermediary group, formed by T-Coffee, Muscle, Mafft FFT-NS-2 and Kalign in some cases presented similar results to the top two algorithms, especially T-Coffee and Mafft FFT-NS-2 in tests with short evolutionary distances and low gap frequency and length. This showed the quality of the algorithms and that different approaches to sequence alignment can converge on a very similar MSA.

Additionally, we only tested the programs with their default parameters; different program configurations might improve their accuracy. Our results are consistent with those previously reported in the original articles of Mafft L-INS-i [25] and ProbCons [28], where they ranked top with the best accuracy on BAliBASE v2 alignments.

In this work, it could be observed that all programs have strengths and weaknesses, and among the best performers Mafft has the most flexible algorithm. The recent additions to the program certainly contributed to improve alignment accuracy. Mafft also has a very fast algorithm even when aligning iteratively. It has been suggested that Mafft's accuracy could be increased by incorporating structural information [25]. ProbCons had very similar results and sometimes performed even better than Mafft L-INS-i, but it is the second slowest program overall. The alignment power of its algorithm is excellent, even though it does not consider any biological aspect of the sequences when performing an MSA.

In the intermediary group, T-Coffee and Muscle were the better alternatives, considering that Mafft FFT-NS-2 did not perform as well as the iterative approaches, and Kalign showed inconsistent results in most cases faring below the other three programs. T-Coffee generates good alignments and has the merit of combining alignments from different sources [25], but the processing time is the worst for every sequence size. Muscle, on the other hand, is an iterative program that produces good quality alignments, often comparable to T-Coffee and Mafft FFT-NS-2, with the advantage of being extremely fast. Muscle allows an increase in the number of iterative steps in its procedure (not tested here) that can probably ameliorate its final alignment quality. Kalign presented accuracy values in most of the cases lower than the other three programs in this intermediary group, but showed very good results at low indel frequency values. The packages with poorest performance, Clustal W, POA, Dialign-T and Dialign2.2, also present qualities such as the rapid assembly of accurate MSAs of closely related sequences with a low number of indels. These programs may be employed to create an initial alignment that can be further improved with another algorithm. Clustal W showed good accuracy results in the alignment of short sequences with indels, but had a steady decline when the length of the sequence containing indels was increased. Although Dialign-T developers claim that the program's new implementation generates better results than version 2.2, this could not be seen in our results. In the simulated sequence analysis, Dialign-T was inconsistent, sometimes as accurate as Clustal W, while otherwise comparable to or worse than Dialign2.2. Dialign-T's accuracy was originally tested solely on alignment databases (BAliBASE v2.1 and IRMbase) [29]. When evaluated against a more diverse collection of protein sequences one can see that the program does not fare as well as claimed initially.

Apparently, programs that have a tree-building step in their alignment procedure seemed to produce better results than programs that do not build a phylogenetic tree or cluster in their alignment process. Of the bottom four performers, only Clustal W builds a Neighbor Joining tree to guide the multiple sequence alignment. According to POA developers, their program is more suited for alignment of multidomain sequences, and a way to improve the algorithm would be to use a Clustal-like progressive alignment with a guide tree [20]. Also, our results demonstrate that POA had the tendency to insert large gaps on the sequences' terminal regions, which inflated the average number of gaps per sequence. This led to the low accuracy values generated by POA and a visual inspection of a considerable number of resulting sets revealed that the intermediary regions of the alignments were consistent with other packages results. It was also shown before that global alignment programs usually perform better than local alignment algorithms such as Dialign2.2 and Dialign-T [33]. These two programs seemed to be more suitable to align sequences with high local similarities that were shuffled by recombination. Both programs were among the least conservative in inserting gaps, which may explain the low alignment accuracy values obtained.

Among the programs that use a guide-tree in their multiple alignment procedure, hierarchical clustering outperformed UPGMA with modified linkage (in Mafft's non-iterative approach), Neighbor Joining and UPGMA (Muscle and Kalign). Clustal W had the lowest accuracy in the group but in many cases it outperformed the programs that lack tree-building capabilities, probably because of its profile alignment procedure. Kalign, that uses UPGMA (Neighbor Joining is an option) had superior results in comparison to Clustal W, what might be explained by the distinct algorithm that calculates pairwise distances. T-Coffee showed better accuracy than Clustal W and Kalign, maybe because of the incorporation of Lalign alignments in its algorithm, that improved the pairwise alignment generated by a Clustal W-like process. At the same time, Muscle performed as well as T-Coffee and Mafft FFT-NS-2, showing that all tree-building methods might be equivalent. UPGMA with modified linkage had an edge when the iterative capabilities of Mafft were employed. Finally, hierarchical clustering, which does not incorporate biological concepts in its calculation, was better than the biology-based tree determination methods in some the tested scenarios.

Regarding factors that influence alignment accuracy, indel number is surely the one with the largest effect. The overall performance of all programs decreased proportionally to the increase in gap frequency and to a lesser extent indel size. This was shown by increasing indel frequency alone, indel length alone and both combined. Among these two parameters, indel number seems to have more consequences to accuracy loss than indel length alone, maybe because most alignment algorithms have a tendency to merge gaps. Larger evolutionary distance plays a role in the quality of MSA, and this might be related to an increased number of indel events with longer branch lengths. In cases with both low indel frequency and length, sequences were aligned by all packages accurately even for simulations based on trees with long branches. These results show that some alignment programs tend to be conservative with respect to inserting gaps as the loss of accuracy is mainly due to inferred alignments having fewer and shorter gaps than the known alignments. Although different programs have distinct allowances for terminal gaps we showed that terminal gaps did not have a large effect on alignment accuracy, regardless of their length.

## Conclusion

Our analysis reveals that Mafft is the best choice for protein sequence alignment, based on its overall alignment quality and processing speed. Other algorithms, however, cannot be dismissed as they showed very good results for some evolutionary scenarios. By comparing accuracy and date of publication of the programs (Figure 1), it seems that overall alignment quality has generally improved over time, but there is still room for improvement as alignment accuracy is still fairly low in many cases.

With the advent of Simprot, there is another alternative to assess MSA performance. Our study shows that Simprot and simulated sequences present a reliable approach to check alignment quality. This methodology proved to be more flexible and able to generate a broader range of alignment classes in comparison to methods used in the past. Although the final conclusions were similar while using our method and BAliBASE sets of protein alignments, our methodology allows us to determine with more detail the strengths and weaknesses of each alignment program and its algorithmic approach. Simprot also proved to be a suitable alternative for alignment quality testing. In conclusion, the ability to create large simulated alignment sets in seconds, with full control of its characteristics, allows a quick and reliable analysis of different evolutionary histories, some of them not available in the current database sets.

## Methods

### BAliBASE

All five BAliBASE data sets, including sub-references, were aligned using the nine programs described above and the obtained alignment compared to the original alignment file provided. Accuracy was measured for each alignment and the average of each program was compared separately for each reference and overall for the whole database. A Wilcoxon signed ranks test was used to assess statistical significance of the results.

### Simprot simulated sequences

Simprot was used to simulate sets of protein sequences in different evolutionary conditions. This simulation program requires a phylogenetic tree as its initial input in order to generate a file with the known alignment which is determined from the known evolutionary history of the sequences. In this study, we used five different bifurcating trees, attempting to include general scenarios of evolution with distinct topologies and characteristics in trees obtained from protein MSA and trees created artificially (Figure 2). Two of these trees, tree B (44 taxa) and tree C (20 taxa) were obtained based on PFAM alignments [34]. Artificial trees, D and E, both with 16 taxa, had identical topologies and maximum evolutionary distance, differing on when and where evolution had occurred. Finally, tree A also contained 16 taxa in a bifurcating topology, with a large monophyletic clade of 12 taxa and a small sister clade of four taxa. All branches were of equal length except at the node that supported the larger clade, which was twice the size of the other branches.

### Alignment accuracy evaluation

Another critical step when comparing the results from different algorithms is the alignment scoring. There are many scoring functions available. BAliBASE creators also have introduced two distinct scores for the comparison of an alignment against a reference set: column score (CS) and sum-of-pair score (SP) [33]. The SP score is defined as the number of correctly aligned residues pairs that are found in the test alignment divided by the total number of aligned residue pairs of the reference alignment, increasing with the number of sequences aligned correctly. SP calculation takes into account pairs of aligned residues occurring in both MSA, while the CS calculation only checks for identical columns in each set of aligned sequences. SP is also known as *f*_*D*_, the developer's score [35,36]. A third option is the modeler's score (*f*_*M*_) which indicates the fraction of residue pairs in the test alignment that are correctly aligned in comparison to the reference [37].

Alignment accuracy, either for BAliBASE sequences or Simprot simulated sequences, was measured using the developer's score and the modeler's score [35]. Both scores are calculated as

fD=cr,fM=ct
 MathType@MTEF@5@5@+=feaafiart1ev1aaatCvAUfKttLearuWrP9MDH5MBPbIqV92AaeXatLxBI9gBaebbnrfifHhDYfgasaacH8akY=wiFfYdH8Gipec8Eeeu0xXdbba9frFj0=OqFfea0dXdd9vqai=hGuQ8kuc9pgc9s8qqaq=dirpe0xb9q8qiLsFr0=vr0=vr0dc8meaabaqaciaacaGaaeqabaqabeGadaaakeaacqWGMbGzdaWgaaWcbaGaemiraqeabeaakiabg2da9maalaaabaGaem4yamgabaGaemOCaihaaiabcYcaSiabdAgaMnaaBaaaleaacqWGnbqtaeqaaOGaeyypa0ZaaSaaaeaacqWGJbWyaeaacqWG0baDaaaaaa@3A7E@

where *c *number of residue pairs in the test alignment that are correctly aligned with respect to the reference alignment, *r *number of aligned residue pairs in the reference alignment and *t *number of aligned residue pairs in the test alignment. Both scores have a maximum value of 1 (all pairs correctly aligned), and a minimum equal to 0 (no pairs are correctly aligned). The developer's and modeler's scores have been the most widely used in alignment score assessment and were featured in a comparison of profile alignment scoring by Edgar and Sjölander [38].

The two alignment scoring functions tested yielded very similar results. In most cases, the modeler's score resulted in a value slightly lower than the developer's score, while in very few cases a modest improvement was observed. We therefore decided to present here only the results obtained using the developer's score.

### Performance evaluation

All programs' performance was also tested in aligning sequences of different sizes. We did not evaluate the speed variation regarding the number of input sequences, due to restrictions in some programs in aligning large numbers of sequences. Using Simprot's default parameters and tree B (44 taxa) as input, ten sets of simulated protein sequences were generated in seven lengths: 100, 200, 300, 400, 500, 1000 and 1500 amino acids. Total CPU time, calculated by the system *time *command was averaged. All programs were run in a dual 3.0 Ghz Xeon with 4 GB of memory, running openMosix with Linux kernel 2.4.22.

## Abbreviations

CS: column score

FFT: Fast Fourier Transform

indel: insertion/deletion

MSA: multiple sequence alignment

PO: Partial Order

PO-MSA: Partial Order-Multiple Sequence Alignment

SP: sum-of-pair score

WSP: weighted sum-of-pairs

## Authors' contributions

PASN designed the study, conducted the analysis and drafted the manuscript. ZW wrote the application to measure alignment scores. ERMT participated in the design and coordination of the study and helped to draft the manuscript. All authors have read and approved the final manuscript.

**Table 1:** Factors analyzed in the alignment simulations, related program parameters and values used to simulate the sequences.

Factor	Simprot parameter	Values	Description
sequence length	-r	50, 100, 150, 200, 250, 300	length (in amino acids) of the root sequence
indel frequency	-g	0.03, 0.05, 0.1, 0.15, 0.2, 0.3	expected indel frequency (number of indels/aa) for 100PAM
gamma alpha	-x	0.1, 0.7, 1, 5, 10	shape parameter of the gamma distribution of evolutionary rates
evolutionary scale factor	-c	2, 3, 4	controls the expected length of indels according to the generalized Qian-Goldstein distribution
branch length scale multiplier*	-b	2, 3, 4	scale lengths of all branches in the input tree equally
terminal gaps insertion	not available	1, 2, 3, 4, 5	controls the frequency and lengths of terminal gaps (as a function of internal gap parameters)

* only for tree A (Figure 2)
